# Highly Responsive Gate-Controlled p-GaN/AlGaN/GaN Ultraviolet Photodetectors with a High-Transmittance Indium Tin Oxide Gate †

**DOI:** 10.3390/mi15010156

**Published:** 2024-01-20

**Authors:** Zhanfei Han, Xiangdong Li, Hongyue Wang, Yuebo Liu, Weitao Yang, Zesheng Lv, Meng Wang, Shuzhen You, Jincheng Zhang, Yue Hao

**Affiliations:** 1 Guangzhou Wide Bandgap Semiconductor Innovation Center, Guangzhou Institute of Technology, Xidian University, Guangzhou 510555, China; zfhan@stu.xidian.edu.cn (Z.H.); mengm10612183@163.com (M.W.); youshuzhen@xidian.edu.cn (S.Y.); jchzhang@xidian.edu.cn (J.Z.); yhao@xidian.edu.cn (Y.H.); 2 Key Laboratory of Wide Bandgap Semiconductor Materials and Devices, School of Microelectronics, Xidian University, Xi’an 710071, China; 3 China Electronic Product Reliability and Environmental Testing Research Institute, Guangzhou 511370, China; wanghongyue@pku.edu.cn; 4 China Southern Power Grid Technology Co., Ltd., Guangzhou 510080, China; yangweitao188@163.com; 5 School of Electronics and Information Technology, Sun Yat-sen University, Guangzhou 510006, China; lvzesh@mail.sysu.edu.cn

**Keywords:** p-GaN, indium-tin-oxide (ITO), ultraviolet photodetector (UVPD)

## Abstract

This work presents highly responsive gate-controlled p-GaN/AlGaN/GaN ultraviolet photodetectors (UVPDs) on Si substrates with a high-transmittance ITO gate. The two-dimensional electron gas (2DEG) in the quantum well of the polarized AlGaN/GaN heterojunction was efficiently depleted by the p-GaN gate, leading to a high photo-to-dark current ratio (PDCR) of 3.2 × 10^5^. The quantum wells of the p-GaN/AlGaN and AlGaN/GaN heterojunctions can trap the holes and electrons excited by the UV illumination, thus efficiently triggering a photovoltaic effect and photoconductive effect, separately. Furthermore, the prepared photodetectors allow flexible adjustment of the static bias point, making it adaptable to different environments. Compared to traditional thin-film semi-transparent Ni/Au gates, indium tin oxide (ITO) exhibits higher transmittance. Under 355 nm illumination, the photodetector exhibited a super-high responsivity exceeding 3.5 × 10^4^ A/W, and it could even exceed 10^6^ A/W under 300 nm illumination. The well-designed UVPD combines both the advantages of the high-transmittance ITO gate and the structure of the commercialized p-GaN/AlGaN/GaN high-electron-mobility transistors (HEMTs), which opens a new possibility of fabricating large-scale, low-cost, and high-performance UVPDs in the future.

## 1. Introduction

Gallium nitride (GaN), one of the most well-known wide-bandgap semiconductors, has already been widely applied in various fields such as illumination, power electronics, and communications [1,2]. Recently, GaN has received increased attention for ultraviolet photodetectors (UVPD) [3,4,5,6,7,8,9,10,11,12]. At present, ultraviolet photodetectors are used in aerospace, sensors, flame detection, and other applications. GaN-based photodetectors have been used for metal-semiconductor-metals [13], p-i-n [14], high-electron-mobility transistors (HEMTs) [15,16], avalanche photodiodes [17], and photomultiplier [18] tubes. Compared to bulk materials represented by Si, the heterostructure formed by AlGaN/GaN confines channel electrons near the material surface. This planar structure provides a natural advantage for capturing photos. Therefore, HEMTs with AlGaN/GaN heterostructures exhibit excellent photoresponsivity and photocurrent density, making them more suitable for high-precision optoelectronic detection [13]. However, the presence of the polarization-induced two-dimensional electron gas (2DEG) at the AlGaN/GaN heterostructure induces a high dark current, which jeopardizes the accuracy of detection and induces high-power consumption [19]. One solution is to cut off the 2DEG channel by applying a negative bias to the gate. However, this method may lead to a high off-state current in the dark condition because the negative bias cannot completely deplete the channel electrons. Additionally, designing with negative bias drive can be more complex when integrating designs into circuits. Another solution is to remove the 2DEG under the gate by etching off the AlGaN barrier under the gate [20,21] or by epitaxially growing a p-GaN layer on top of the AlGaN/GaN heterostructure [22,23]. Etching the AlGaN layer requires high stability in the etching process, and the process window is narrow, which makes it challenging to achieve large-scale production. It is worth mentioning that p-GaN/AlGaN/GaN HEMTs have achieved great success in the field of power electronics and have been successfully commercialized [24,25]. Considering that the device structure is almost the same, and in the case of high-energy ion implantation isolation, the leakage current of the device can be in the pA range, which is just conducive to the application of photodetectors in the field of low light intensity detection, and the lower off-state current will undoubtedly reduce the detection interference and improve the sensitivity of the detector, it is possible for them to be widely used in photodetectors. To improve the sensitivity, ITO was deposited on top of the AlGaN layer as the gate material with a high light transmittance for the AlGaN/GaN HEMT photodetectors [26]. Compared to the floating p-GaN ultraviolet photodetector, this structure with a transparent gate allows for the flexible selection of gate voltage, enabling the detector to operate under more ideal bias conditions; this is significant for the robustness of the detector in various environmental conditions. However, ITO-gated p-GaN/AlGaN/GaN photodetectors have not yet been reported that can realize a high photoresponsivity by combining high transmittance and low dark current.

In this work, we present the development of ITO-gated p-GaN/AlGaN/GaN photodetectors on large-scale Si substrates. The epitaxial structure of p-GaN/AlGaN/GaN on Si is identical to that used in power electronics applications, with the sole exception of the transparent ITO gate. We will evaluate the performance of these detectors under various wavelengths and intensities of light and compare them with similar structured detectors. Some of the measuring results from this study have already been presubmitted to a conference [27]. Furthermore, in this work, we will delve into the mechanisms behind achieving high responsivity in the ITO/p-GaN/AlGaN/GaN photodetector and provide an explanation for the formation of photocurrent from a band structure perspective.

## 2. Experiment and Methods

**Figure 1:**
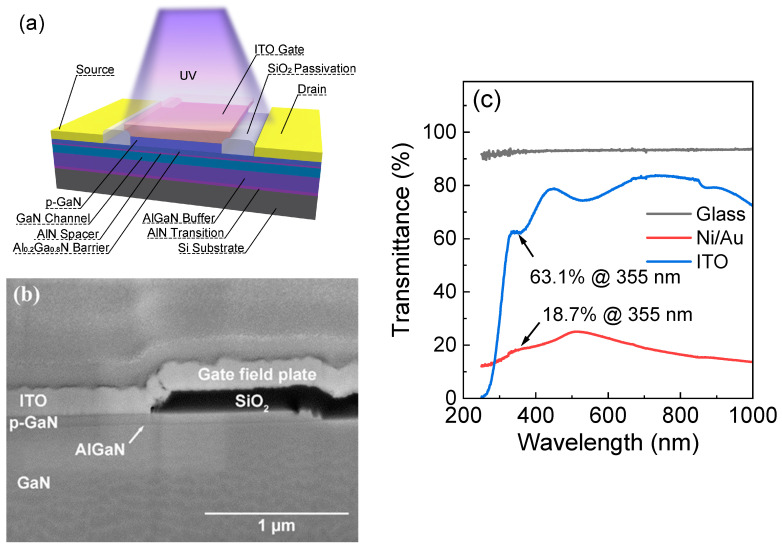
(**a**) Schematic structure and (**b**) SEM photo of the UV photodetector with a transparent ITO gate in the p-GaN/AlGaN/GaN heterostructure (above the ITO gate electrode, protective platinum metal has been deposited prior to FIB). (**c**) Transmittance curves of Ni/Au and ITO for light wavelengths on a high-transmittance glass.

The employed heterostructure is a standard configuration in p-GaN gate high-electron-mobility transistors (HEMTs) and was epitaxially grown on a 6-inch Si <111> substrate using metal-organic chemical vapor deposition (MOCVD). The epitaxial structure, grown from bottom to top, comprised a 4 µm AlGaN buffer layer, a 200 nm GaN channel layer, a 0.7 nm AlN insertion layer, a 15 nm Al_0.2_Ga_0.8_N barrier layer, and a 70 nm Mg-doped p-GaN layer with a doping concentration of 3 × 10^19^ cm^−3^. After the epitaxial process, the p-GaN layer was activated at 750 °C for 10 min in the MOCVD chamber. During the activation process, the Mg elements, which were passivated by H, became effective acceptor impurities. The activated p-GaN can efficiently cut off the channel, which is a well-established technique in the field of power electronics [28]. The device structure is illustrated in Figure 1a,b shows a cross-section of the gate region of the device.

Figure 1c demonstrates light transmittance characteristics. The thin layer of Ni/Au deposited on the high-transmittance glass shows only 18.7% transmittance under the illumination of a 355 nm wavelength. In contrast, ITO on the same substrate exhibits a significantly higher transmittance of 63.1%. This observation underscores the advantage of using ITO as a transparent gate electrode material on GaN, ensuring a more effective photon interaction for generating photoelectric effects. The ITO used in this work was deposited using an optical coating machine at a substrate temperature of 350 °C. The high-temperature deposition was employed to ensure better transparency but excessively high temperatures can lead to an increase in its resistivity [29].

The length of p-GaN (L_G_) in the device prepared in this work is 6 µm, gate-source spacing (L_GS_) is 1.5 µm, gate-drain spacing (L_GD_) is 1.5 µm, and the active area width (W_G_) is 100 µm. The gate’s absorbing area is 6 µm × 100 µm. The schematic representation of the device structure is provided in Figure 1a.

The fabrication process began with inductively coupled plasma (ICP) dry etching, using a Cl_2_/Ar/O_2_ gas mixture for selectively removing p-GaN, which was crucial to the successful fabrication of the device, and achieving a selection ratio of more than 40:1, which resulted in a roughness of 0.5 nm in the AlGaN stop layer. The roughness was obtained via atomic force microscopy (AFM). Afterwards, a metal stack consisting of Ti/Al/Ni/Au (20/140/50/40 nm) was deposited using electron beam evaporation, followed by a 30-s annealing step at 865 °C in a nitrogen environment to create Ohmic contacts. The thickness of the metal stack and annealing parameters were extensively validated in power electronic devices [30]. A 200 nm SiO_2_ passivation layer was then deposited with plasma-enhanced chemical vapor deposition (PECVD) at 350 °C. Device isolation was achieved through multiple-energy N-ion implantation. The passivation layer on top of the p-GaN was selectively removed using reactive ion etching (RIE) to create the gate window. An ITO layer with a thickness of 225 nm was deposited using an optical coater. Finally, ion beam etching (IBE) was utilized for patterning the ITO gate, and RIE was performed to open the source-drain windows.

## 3. Results and Discussion

**Figure 2:**
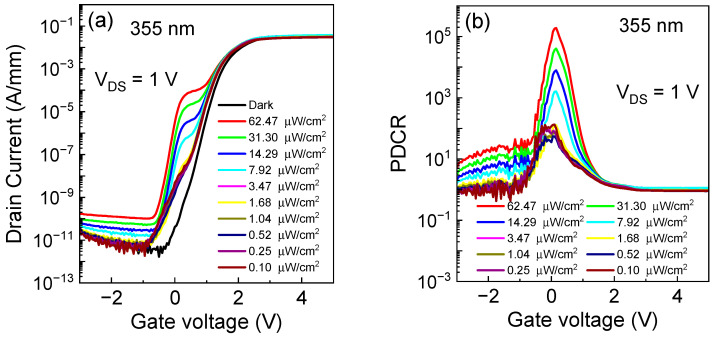

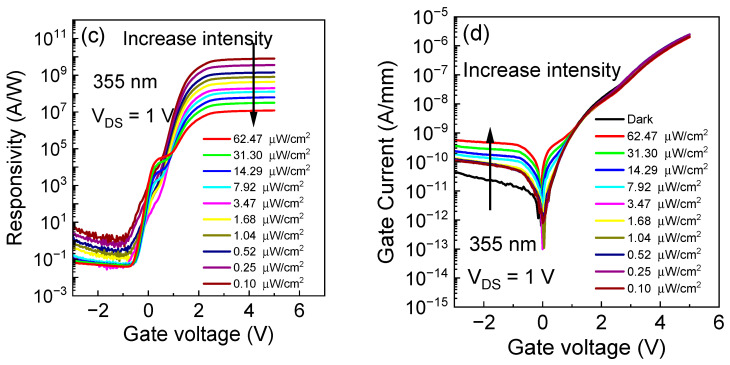
(**a**) Transfer characteristics, (**b**) photo-to-dark current ratio (PDCR) and (**c**) responsivity of the device at different illumination intensities of V_DS_ = 1 V. (**d**) Gate leakage current curves at different illumination intensities and a wavelength of 355 nm.

In Figure 2a, the transfer characteristics of the photodetector are analyzed under 355 nm light exposure at different levels of illumination intensity. The dark-state threshold voltage (V_TH_) of the photodetector, determined by the criterion I_DS_ = 0.1 mA/mm, is around 1.2 V. As observed in Figure 2a, there is a plateau near the threshold voltage. This phenomenon occurs because the influence of illumination on the drain current is more significant before the dark-state threshold voltage for lower light intensities or lower illumination intensities. Once the gate voltage exceeds the dark-state threshold voltage, the device primarily opens under the control of the gate voltage. Additionally, the saturation current of the photo-controlled device is significantly smaller than that of the device controlled by gate voltage. Therefore, when the gate voltage is below the threshold voltage, the saturation of the photo-controlled current leads to the appearance of the platform. Figure 2b depicts the variation of photo-to-dark current ratio (PDCR) with V_GS_ across different levels of illumination intensity. A pronounced peak in PDCR occurs at V_GS_ = 0.15 V, reaching a maximum value of 1.9 × 10^5^, corresponding to a light power intensity of 62.47 µW/cm^2^. When the gate voltage is below −1 V, the device is primarily influenced by the gate voltage, and ultraviolet light cannot effectively open the channel that is closed by the gate. By examining Figure 2a,d together, we can observe that, in this case, the drain current is mainly caused by gate leakage. When the gate voltage is greater than the threshold voltage in the dark state, the device mainly operates under the control of the gate voltage. The magnitude of the photocurrent under low light intensity is much smaller than the conduction current under the HEMTs’ gate control, so its light-to-dark current ratio tends towards 1. In Figure 2c, the responsivity is compared with V_GS_ at different illumination intensities. A sharp increase in responsivity is observed at V_GS_ = −0.5 V, such as the behavior seen in the V_TH_ of the transfer characteristics in Figure 2a. Figure 2d presents the gate leakage current under ultraviolet light illumination. The reverse gate leakage current experiences a more significant increase under illumination compared with the forward gate leakage current. This phenomenon is rational because the reverse gate leakage current is limited by the reversely biased p-GaN/AlGaN/GaN p-i-n junction, where photon-generated carriers in the depletion region easily enhance reverse leakage. As the gate bias surpasses 1 V, the forward gate leakage gradually transitions to being dominated by hole injection from the gate metal into the p-GaN layer [31]. In this case, the junction formed by p-GaN and ITO is under reverse bias, and the influence of light illumination on the leakage is almost negligible because of the large leakage current when Schottky works in the dark state.

**Figure 3:**
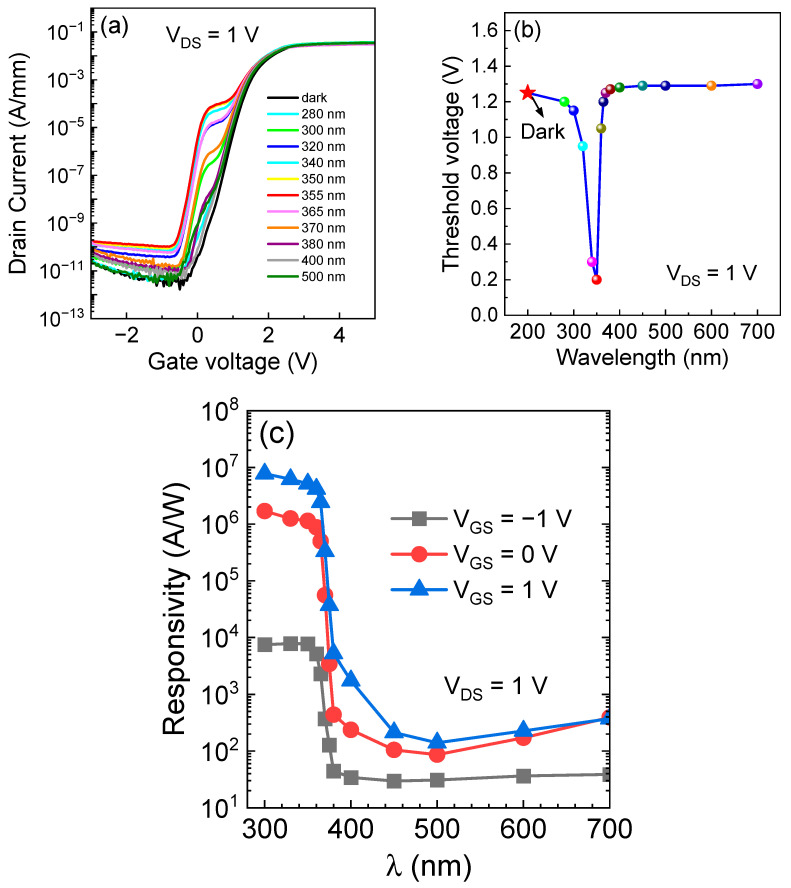
(**a**) Transfer characteristics and (**b**) threshold voltage distribution of the device at versus wavelength at V_DS_ = 1 V, (**c**) response curves at different wavelengths under V_GS_ = −1/0/1 V conditions.

**Figure 4:**
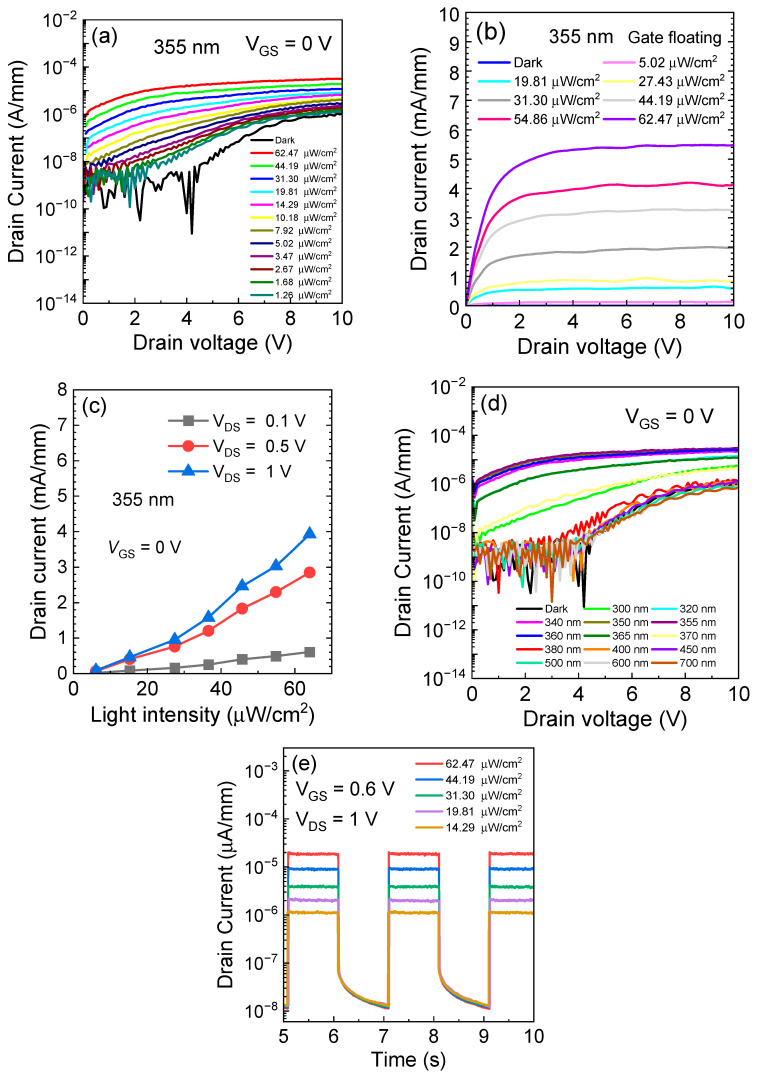
(**a**) Semi-logarithmic curves (V_GS_ = 0 V) and (**b**) linear curves of the output characteristics (gate floating) of a 355nm light source at different illumination intensities. (**c**) Curves of drain currents at different illumination intensities under V_DS_ = 0.1/0.5/1 V conditions. (**d**) Output characteristics of different wavelengths. (**e**) Transient response of UVPD at V_GS_ = 0.6 V, V_DS_ = 1 V.

Further, we measured the transfer characteristics of UVPD at different wavelengths. Figure 3a,b shows the threshold voltage (@ I_DS_ = 0.1 mA/mm) at different wavelengths. The results show that the threshold voltage around 355 nm decreases rapidly because of the influence of ultraviolet light near the subthreshold. This decrease is attributed to the Schottky junction formed by ITO and p-GaN operating in a reverse-biased state, and the depletion region within p-GaN is affected by the illumination, generating more non-equilibrium charge carriers. In the p-GaN/AlGaN/GaN p-i-n junction, the non-equilibrium holes generated by absorbing photons accumulate at the AlGaN/p-GaN interface. On the one hand, because of electrostatic equilibrium, these non-equilibrium holes induce the accumulation of electrons at the AlGaN/GaN interface; on the other hand, the accumulated charge forms a photo-induced electric field, reducing the barrier. Additionally, the illumination causes the separation of electron-hole pairs in the GaN. These pairs experience the influence of the polarization electric field, leading to premature device conduction. Figure 4a,d display the output characteristics at various illumination intensities and different wavelengths of light. At a wavelength of 355 nm, I_DS_ exhibits a four-order magnitude increase, subsequently returning abruptly to the dark current level. This behavior can be explained using the following formula:

(1) $$E=\frac{hc}{\lambda }=5.4\times 10^{-19}J=3.49\;eV$$

where *E* is the energy of a single photon, *h* is Planck’s constant, *c* is the speed of light in a vacuum, and *λ* is the wavelength 355 nm of the incident light, corresponding to the energy of 3.49 eV that is indeed around the band gap energy *E_g_* of GaN materials [32].

When ultraviolet light is within a wavelength greater than 355 nm, electrons are unable to cross the bandgap, thus having no influence on the drain current. From Figure 3b, we can observe that as the wavelength increases, the threshold voltage quickly returns to dark conditions. Additionally, with the gradual increase in wavelength towards the infrared spectrum, the threshold voltage tends to drift positively. This might be attributed to the increase in temperature, which reduces the device’s current, leading to an artificially higher threshold voltage extracted through the constant current method. For UV light with wavelengths below 355 nm, the photocurrent is smaller. We attribute this limitation to limitations imposed by the equipment used. When fixing the slit width, the photon flux for shorter-wavelength light is reduced, leading to a lower actual power density reaching the device surface. To facilitate the comparison of responses at different wavelengths, the results were normalized, as shown in Figure 3c. Notably, a significant responsivity change was observed around 355 nm, in excellent agreement with previously derived theoretical results. The results clearly indicate that, for ultraviolet light around 300 nm, the responsivity can reach up to 10^6^ A/W at V_GS_ = 0 V.

Measurements were conducted to further characterize the output characteristics of the ultraviolet (UV) photodetector; the measurements were conducted using different intensities of a 355 nm UV light source, as depicted in Figure 4a,b. With V_GS_ maintained at 0 V, the UV illumination demonstrated output characteristics resembling those of gate voltage-controlled devices. The photocurrent increased with the increase in light intensity, and it saturated around a drain voltage of 2 V. Figure 4c illustrates the photocurrent under different light intensities at V_DS_ = 0.1/0.5/1 V conditions. It is evident that the photocurrent increases almost linearly with the increment of light intensity, indicating the good linearity of the fabricated photodetector.

Figure 4d illustrates output characteristics at various wavelengths. It can be observed from the graph that there is only a noteworthy photocurrent response for light sources near 355 nm. For ultraviolet light sources with shorter wavelengths, the photocurrent is not ideal. This observation can be attributed to the fact that, when controlling the same slit width, shorter-wavelength light has a lower probability of passing through the slit, thereby leading to a lower actual illumination intensity.

To further analyze the photodetection performance, transient measurements were conducted at V_GS_ = 0.6 V and V_DS_ = 1 V. The current and time curves under 355 nm illumination at different intensities are shown in Figure 4e. It is observed that the UVPD (ultraviolet photodetector) exhibited a rapid response and generated photocurrent within milliseconds in saturation mode. The 10–90% rise time and 90–10% fall time were extracted as 3.5 ms and 4.9 ms, respectively, indicating favorable photodetection characteristics for the high-electron-mobility transistor (HEMT) structure. However, it is important to note that the presence of interface defects on the AlGaN surface may lead to electron capture within the channel, causing a delay in the detector’s response to changes in illumination.

Table 1 summarizes the key performance of several detectors of similar construction. The detector prepared in this work achieves a detection rate of 3.5 × 10^4^ A/W at a gate voltage of 0.6 V, and the low dark current of the 3 × 10^−9^ A/mm is competitive with other similar detectors. Compared to ultraviolet photodetectors without gate metal, the detectors in this work offer the advantage of being able to choose static bias voltages, enabling higher robustness to adapt to different environments. The response rate for ultraviolet light is near 300 nm and responsivity can reach as high as 10^6^ A/W, which provides a highly competitive edge among similar products in the market. Compared to the p-GaN HEMT structure with a thin Ni/Au gate metal layer, our detectors achieved higher responsivity, primarily attributed to the higher transparency of ITO; this allowed more photons to pass through the transparent gate and be captured, leading to a significant advantage for low-light detection. Compared to detectors without a p-GaN gate, the devices in this work achieved lower dark current and a higher ratio of photocurrent to dark current. Furthermore, because of positive biasing, we significantly simplified the design complexity of the gate-driving portion in integrated optoelectronic circuits.

**Figure 5:**
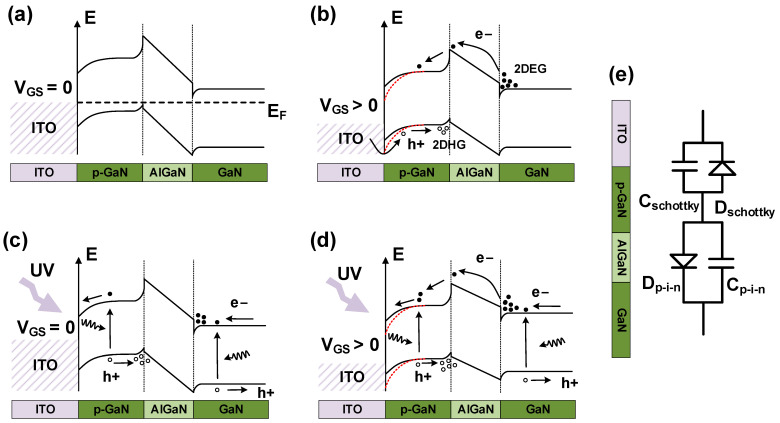
Energy band diagram of p-GaN/AlGaN/GaN heterostructure in (**a**) thermal equilibrium state at V_GS_ = 0 V, (**b**) under positive gate bias, (**c**) under ultraviolet illumination at V_GS_ = 0 V, and (**d**) under UV illumination with positive gate bias. (**e**) Equivalent model of the gate stack.

Next, to elucidate the mechanism of the ITO-gated p-GaN/AlGaN/GaN UVPD that is behind the remarkable responsivity achieved, band diagrams of the device under various conditions are presented in Figure 5. In the equilibrium state without any light illumination, as shown in Figure 5a, the built-in electric field between ITO/p-GaN tilts the bands in the upper surface p-GaN significantly. On the one hand, the width of the depletion region increases, and the electron holes in the depletion region are easier to absorb photons and separate to the neutral region, thereby greatly increasing the number of photons absorbed by the p-GaN surface. On the other hand, the built-in electric field also prevents the holes in the p-GaN from diffusing into the ITO.

Figure 5b shows the band diagram under a positive gate bias. Under the action of positive potential, holes cross the Schottky barrier from the ITO into the p-GaN and are accumulated in the quantum well at the p-GaN/AlGaN interface and form a two-dimensional hole gas (2DHG), while others are lost through the AlGaN barrier into the GaN channel. The 2DHG is necessary to induce the 2DEG channel via electrostatic coupling in the AlGaN/GaN quantum well [33]. As shown in Figure 5c, under 355 nm illumination, the electron-hole pairs generated in p-GaN and AlGaN are easily separated by polarized electric fields, resulting in the formation of a 2DEG, which negatively shifts the V_TH_ and turns on the device in the subthreshold region. Additionally, the accumulated carriers create an optically generated electric field, which reduces the extent of the degree of band tilting in the barrier layer. Figure 5d exhibits a band diagram where the simultaneous application of gate bias and UV light illumination is depicted. The initial gate voltage primarily drops across the AlGaN/GaN heterojunction to attract more electrons to accumulate in the AlGaN/GaN quantum well. Simultaneously, the introduction of light causes the separation of electron-hole pairs. Electrons, having gained photon energy and transitioned to the conduction band, rapidly flow into the gate under a smaller positive gate voltage. At the same time, holes accumulate at the p-GaN/AlGaN interface, inducing an equal number of electrons in the AlGaN/GaN region. These electrons are confined within the quantum well, forming a conductive channel. As the gate voltage further increases, the 2DEG gradually saturates and the increased gate voltage turns to drop on the ITO/p-GaN heterostructure, inducing the band diagram to bend.

Figure 5e shows the equivalent circuit model of the gate stack that can be roughly modeled as two back-to-back diodes. Under ultraviolet light illumination, electron-hole pairs are generated in p-GaN, and many non-equilibrium holes are injected into ITO from p-GaN when the ITO gate is reversely biased, which explains the pronounced gate leakage increase in Figure 2d.

## 4. Conclusions

In conclusion, we have successfully demonstrated a highly responsive ITO-gated p-GaN/AlGaN/GaN ultraviolet photodetector (UVPD). Leveraging the p-GaN/AlGaN/GaN structure and the transparent ITO gate, we achieved an ultra-low dark current of 3 nA/mm, a high photo-to-dark current ratio (PDCR) of 1.9 × 10^5^, and a high responsivity exceeding 3.5 × 10^4^ A/W. Moreover, with the introduction of the high-transparency ITO gate, the static bias voltage of the detector can be flexibly modified by applying an external gate voltage, thereby achieving enhanced characteristics. Finally, from the perspective of energy bands, the reasons for the formation of high responsivity to ultraviolet light are analyzed in depth. This research has introduced a novel approach for producing high-performance, cost-effective UVPDs that are compatible with the existing GaN electronic industry, thereby contributing to the advancement of UVPD technology.

## Figures and Tables

**Table 1:** Comparison of key performance of ultraviolet photodetectors with different structures based on AlGaN/GaN heterojunctions.

Structure	λ (nm)	V_GS_/V_DS_ (V)	R (A/W)	I_dark_ (A/mm)
Metal/Al_0.45_Ga_0.55_N/Al_0.3_Ga_0.7_N [6]	280	−2/5	6.2 × 10^4^	8.0 × 10^−5^
Metal/Al_0.3_Ga_0.7_N/GaN [7]	365	−8.2/8	1.0 × 10^6^	2.0 × 10^−9^
ITO/Al_0.26_Ga_0.74_N/GaN [9]	360	−3.1/6	2.0 × 10^5^	3.0 × 10^−9^
p-GaN/Al_0.2_Ga_0.8_N/GaN [10]	365	0/5	2.0 × 10^4^	1.0 × 10^−9^
p-GaN/Al_0.2_Ga_0.8_N/GaN [11]	345	0/5	6.8 × 10^4^	4.84 × 10^−10^
Au/Ni/p-GaN/Al_0.2_Ga_0.8_N/GaN [8]	310	0.68/5	6.1 × 10^3^	5.2 × 10^−10^
This work	355	0.6/1	3.5 × 10^4^	3 × 10^−9^
This work	300	0/1	2.0 × 10^6^	3 × 10^−9^

## Data Availability

Data is contained within the article.

## References

[1] Wu Y.F., Gritters J., Shen L., Smith R.P., Swenson B. (2013). kV-class GaN-on-Si HEMTs enabling 99% efficiency converter at 800 V and 100 kHz. IEEE Trans. Power Electron..

[2] Roccaforte F., Greco G., Fiorenza P., Iucolano F. (2019). An overview of normally-off GaN-based high electron mobility transistors. Materials.

[3] Aggarwal N., Gupta G. (2020). Enlightening gallium nitride-based UV photodetectors. J. Mater. Chem. C.

[4] Alaie Z., Nejad S.M., Yousefi M.H. (2015). Recent advances in ultraviolet photodetectors. Mater. Sci. Semicond. Process..

[5] Baek S.-H., Lee G.-W., Cho C.-Y., Lee S.-N. (2021). Gate-controlled amplifiable ultraviolet AlGaN/GaN high-electron-mobility phototransistor. Sci. Rep..

[6] Armstrong A.M., Klein B., Allerman A.A., Douglas E.A., Baca A.G., Crawford M.H., Pickrell G.W., Sanchez C.A. (2018). Visible-blind and solar-blind detection induced by defects in AlGaN high electron mobility transistors. J. Appl. Phys..

[7] Zhang H., Liang F., Song K., Xing C., Wang D., Yu H., Huang C., Sun Y., Yang L., Zhao X. (2021). Demonstration of AlGaN/GaN-based ultraviolet phototransistor with a record high responsivity over 3.6 × 107 A/W. Appl. Phys. Lett..

[8] Chen D., Chen Y.-C., Zeng G., Li Y.-C., Li X.-X., Peng B.-F., Lu H.-L. (2022). Comprehensive analysis of optoelectronic performance of ultraviolet phototransistors based on AlGaN/GaN heterostructure. Semicond. Sci. Technol..

[9] Narita T., Wakejima A., Egawa T. (2013). Ultraviolet photodetectors using transparent gate AlGaN/GaN high electron mobility transistor on silicon substrate. Jpn. J. Appl. Phys..

[10] Lyu Q., Jiang H., Lau K.M. (2020). High gain and high ultraviolet/visible rejection ratio photodetectors using p-GaN/AlGaN/GaN heterostructures grown on Si. Appl. Phys. Lett..

[11] Wang H., You H., Xu Y., Sun X., Wang Y., Pan D., Ye J., Liu B., Chen D., Lu H. (2022). High-responsivity and fast-response ultraviolet phototransistors based on enhanced p-GaN/AlGaN/GaN HEMTs. ACS Photonics.

[12] Chen D., Zhang P., Wang L., Huang W. (2023). Demonstration of p-GaN/AlGaN/GaN-based ultraviolet phototransistors with sub-saturated transfer characteristics. Semicond. Sci. Technol..

[13] Chang S., Chang M., Yang Y. (2017). Enhanced responsivity of GaN metal-semiconductor-metal (MSM) photodetectors on GaN substrate. IEEE Photonics J..

[14] Butun B., Tut T., Ulker E., Yelboga T., Ozbay E. (2008). High-performance visible-blind GaN-based pin photodetectors. Appl. Phys. Lett..

[15] Kuan T.-M., Chang S.-J., Su Y.-K., Ko C.-H., Webb J.B., Bardwell J.A., Liu Y., Tang H., Lin W.-J., Cherng Y.-T. (2003). High optical-gain AlGaN/GaN 2 dimensional electron gas photodetectors. Jpn. J. Appl. Phys..

[16] Li L., Hosomi D., Miyachi Y., Hamada T., Miyoshi M., Egawa T. (2017). High-performance ultraviolet photodetectors based on lattice-matched InAlN/AlGaN heterostructure field-effect transistors gated by transparent ITO films. Appl. Phys. Lett..

[17] Zhou Q., McIntosh D.C., Lu Z., Campbell J.C., Sampath A.V., Shen H., Wraback M. (2011). GaN/SiC Avalanche Photodiodes. Appl. Phys. Lett..

[18] Melton A.G., Burgett E., Xu T., Hertel N., Ferguson I.T. (2012). Comparison of neutron conversion layers for GaN-based scintillators. Phys. Status Scintill..

[19] Hou M., So H., Suria A.J., Yalamarthy A.S., Senesky D.G. (2016). Suppression of persistent photoconductivity in AlGaN/GaN ultraviolet photodetectors using in situ heating. IEEE Electron Device Lett..

[20] Iwaya M., Miura S., Fujii T., Kamiyama S., Amano H., Akasaki I. (2009). High-performance UV detector based on AlGaN/GaN junction heterostructure-field-effect transistor with ap-GaN gate. Phys. Status Solidi C.

[21] Ishiguro M., Ikeda K., Mizuno M., Iwaya M., Takeuchi T., Kamiyama S., Akasaki I. (2013). Control of the detection wavelength in AlGaN/GaN-based hetero-field-effect-transistor photosensors. Jpn. J. Appl. Phys..

[22] Martens M., Schlegel J., Vogt P., Brunner F., Lossy R., Würfl J., Weyers M., Kneissl M. (2011). High gain ultraviolet photodetectors based on AlGaN/GaN heterostructures for optical switching. Appl. Phys. Lett..

[23] Satterthwaite P.F., Yalamarthy A.S., Scandrette N.A., Newaz A.K., Senesky D.G. (2018). High responsivity, low dark current ultraviolet photodetectors based on two-dimensional electron gas interdigitated transducers. ACS Photonics.

[24] Yoshikawa A., Yamamoto Y., Murase T., Iwaya M., Takeuchi T., Kamiyama S., Akasaki I. (2016). High-photosensitivity AlGaN-based UV heterostructure-field-effect-transistor-type photosensors. Jpn. J. Appl. Phys..

[25] Lyu Q., Jiang H., Lau K.M. (2021). Monolithic integration of ultraviolet light emitting diodes and photodetectors on a p-GaN/AlGaN/GaN/Si platform. Opt. Express.

[26] Pu Y., Liang Y.C. (2022). High-gain high-sensitivity AlGaN/GaN ultraviolet photodetector with effective mechanism for photocurrent collection. Appl. Phys. Lett..

[27] Han Z., Li X., Wang H., Liu Y., Wang M., Yuan J., Wang J., Yang W., You S., Zhang J. Transparent ITO gate p-GaN/AlGaN/GaN UV photodetector with high responsivity and high PDCR. Proceedings of the 2023 IEEE Workshop on Wide Bandgap Power Devices and Applications in Asia (WiPDA Asia).

[28] Li J.Z., Lin J.Y., Jiang H.X., Salvador A., Botchkarev A., Morkoc H. (1996). Nature of Mg impurities in GaN. Appl. Phys. Lett..

[29] Kim H., Piqué A., Horwitz J.S., Mattoussi H., Murata H., Kafafi Z.H., Chrisey D.B. (1999). Indium tin oxide thin films for organic light-emitting devices. Appl. Phys. Lett..

[30] Jacobs B., Kramer MC JC M., Geluk E.J., Karouta F. (2002). Optimisation of the Ti/Al/Ni/Au ohmic contact on AlGaN/GaN FET structures. J. Cryst. Growth.

[31] Stockman A., Masin F., Meneghini M., Zanoni E., Meneghesso G., Bakeroot B., Moens P. (2018). Gate conduction mechanisms and lifetime modeling of p-gate AlGaN/GaN high-electron-mobility transistors. IEEE Trans. Electron Devices.

[32] Zhang H., Huang C., Song K., Yu H., Xing C., Wang D., Liu Z., Sun H. (2021). Compositionally graded III-nitride alloys: Building blocks for efficient ultraviolet optoelectronics and power electronics. Rep. Prog. Phys..

[33] Bakeroot B., Stockman A., Posthuma N., Stoffels S., Decoutere S. (2017). Analytical Model for the Threshold Voltage of p-AlGaN High-Electron-Mobility Transistors. IEEE Trans. Electron Devices.

